# Direct Visualization of Arterial Emboli in Moyamoya Syndrome

**DOI:** 10.3389/fneur.2017.00425

**Published:** 2017-08-24

**Authors:** Julie G. Shulman, Samuel Snider, Henri Vaitkevicius, Viken L. Babikian, Nirav J. Patel

**Affiliations:** ^1^Department of Neurology, Boston Medical Center, Boston University School of Medicine, Boston, MA, United States; ^2^Brigham and Women’s Hospital, Department of Neurology, Harvard Medical School, Boston, MA, United States; ^3^VA Boston Healthcare System, Department of Neurology, Boston, MA, United States; ^4^Brigham and Women’s Hospital, Department of Neurosurgery, Harvard Medical School, Boston, MA, United States

**Keywords:** brain ischemia, cerebral revascularization, embolism, moyamoya disease, stroke

## Abstract

**Background:**

Hemodynamic insufficiency is often considered the cause of ischemic stroke in patients with moyamoya syndrome. While high-intensity transient signals (HITS) on transcranial Doppler (TCD) have been reported in this population, the relationship between these signals and ischemic symptoms is not clearly established. Accordingly, current treatment is directed at improving perfusion.

**Clinical presentation:**

We present two patients with symptoms of cerebral ischemia and angiographic findings of moyamoya syndrome. In each case, ischemia may have been caused by either hypoperfusion or embolization. Patient A presented with multifocal right middle cerebral artery (MCA) territory infarctions and angiographic findings consistent with moyamoya disease. She underwent right superficial temporal artery–MCA bypass. Intra-operatively, embolic material was observed and recorded traveling through the recipient MCA branch artery on two occasions. Postoperative TCD demonstrated HITS that resolved with uptitration of antiplatelet therapy. Patient B presented with multifocal, embolic-appearing left MCA infarctions, and unilateral angiographic moyamoya syndrome. She was found to have HITS in the left MCA, which eventually resolved with a combination of antiplatelets and anticoagulation.

**Conclusion:**

Hemodynamic compromise may not be the only cause of brain infarction in patients with moyamoya syndrome. Observations from these two patients provide both direct visualization and TCD evidence of embolization as a potential etiology for brain ischemia. Future investigations into the role of antithrombotic agents should be considered.

## Background

For the last 60 years, neurosurgeons and neurologists have described patients with progressive occlusion of the carotid termini with compensatory proliferation of tiny vessels at the base of the brain. These proliferating vessels, which appear as an early capillary blush or “puff of smoke” on conventional angiogram, lend the moyamoya syndrome its name (1). The moyamoya syndrome refers only to these angiographic findings and can be seen as a consequence of systemic illnesses or treatments, such as Down’s syndrome, atherosclerosis, or cranial irradiation (2). Moyamoya disease refers specifically to idiopathic cases and has been histologically characterized by intimal proliferation, irregular lamina, and medial fibrosis in large arteries, with a marked absence of inflammation. The small proliferative vessels are also atypical and markedly variable in diameter (3).

Patients with moyamoya disease typically present in one of two age groups, either as children or as adults in their 40s, with transient ischemic attack (TIA), stroke, or less frequently, hemorrhage. TIAs and strokes are generally felt to result from proximal stenosis causing hemodynamic insufficiency (4). The possibility of active embolism as a mechanism for cerebral ischemia in moyamoya syndrome has only recently been investigated (5). Understanding the exact mechanism of stroke is important in these patients, as treatment options differ. Hypoperfusion is managed with volume expansion, normocapneia, permissive hypertension, and ultimately surgical bypass. Embolization may be managed with antithrombotic and/or antiplatelet agents (6), which may be risky in fragile moyamoya collaterals.

Transcranial Doppler (TCD) is the primary means of detecting cerebral arterial emboli *in vivo*. Both gaseous and particulate emboli can be identified as high-intensity transient signals (HITS). These signals have a high *in vitro* specificity for emboli (as opposed to artifact) (7), and have been identified in patients with moyamoya disease (8–10). In spite of this, there has never been visual or pathological proof of embolization in these patients.

## Case Presentation

### Patient A

**Figure 1:**
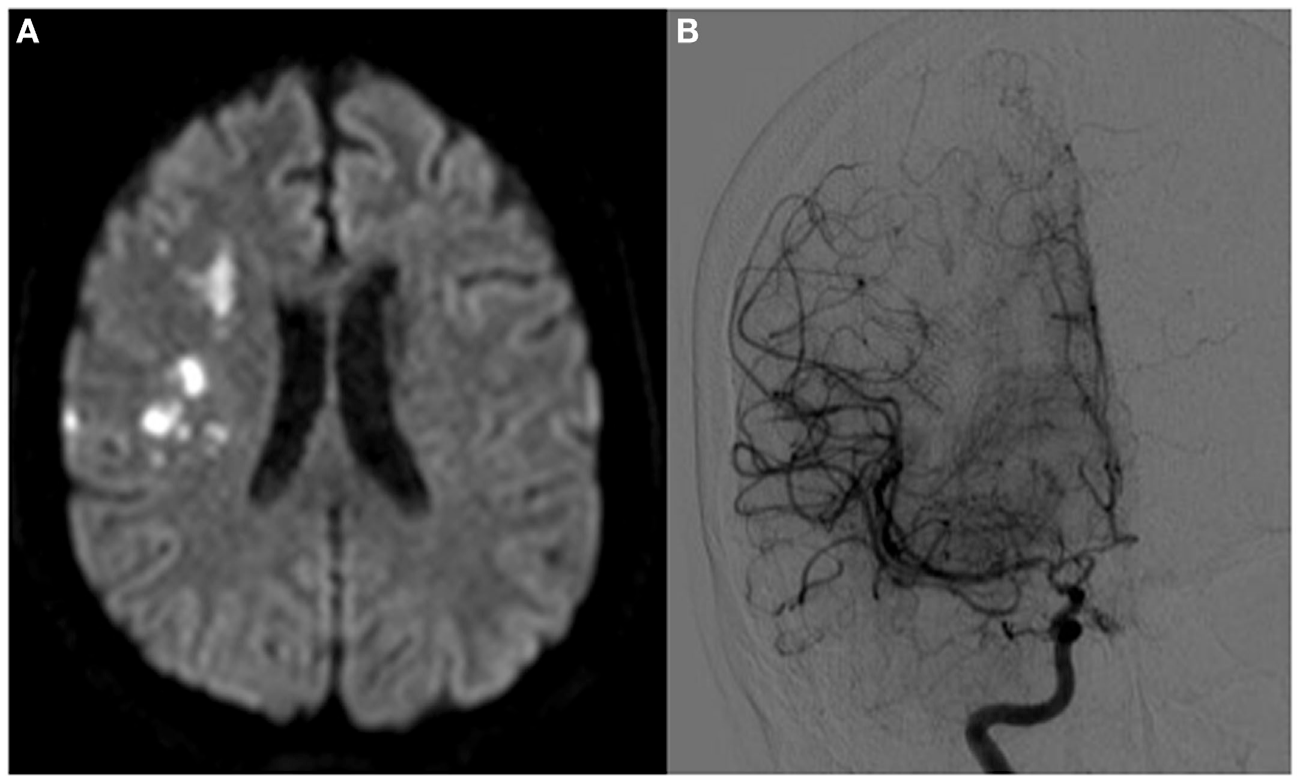
Patient A, a 44-year-old woman with bilateral moyamoya disease, symptomatic on the right side. In panel **(A)**, magnetic resonance imaging demonstrates multiple embolic-appearing ischemic infarcts in the right middle cerebral artery territory. Panel **(B)** demonstrates cerebral angiogram with severe right ICA stenosis with collateralization.

A 44-year-old right-handed hypertensive woman presented with left arm weakness. Symptoms had been present intermittently for several months, but had been particularly severe for 3 days. On examination, she had left hemiparesis and left-sided hyperreflexia. Magnetic Resonance Imaging (MRI) showed acute right middle cerebral artery (MCA) territory infarcts in addition to multiple bilateral chronic infarcts (Figure 1A). Conventional angiogram demonstrated diffusely narrowed bilateral paraclinoid and supraclinoid ICAs, ACAs, and MCAs with numerous collaterals (Figure 1B).

Thorough workup for secondary causes of moyamoya was unremarkable. No hematologic, vasculitic, autoimmune, connective tissue, or genetic abnormalities were identified. A transthoracic echocardiogram showed no central embolic source and no cardiac dysrhythmias were seen on inpatient telemetry monitoring. Given her age, bilateral proximal vasculopathy, and negative secondary workup, she was diagnosed with moyamoya disease. Because of her progressive neurologic symptoms, plans were made to proceed with bilateral direct superficial temporal artery (STA) to MCA bypasses, in stages, beginning with the symptomatic right side.

**Figure 2:**
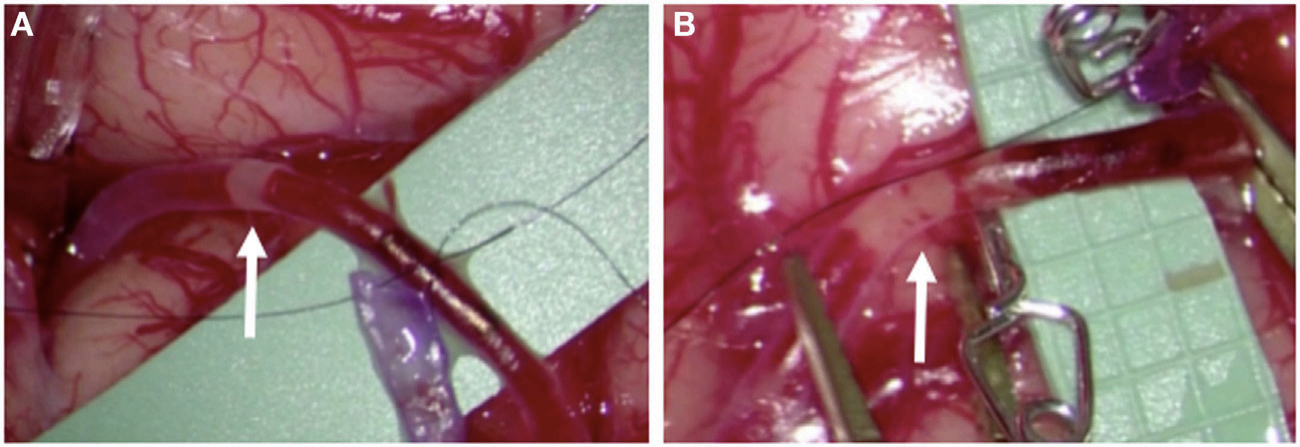
Intra-operative photos taken during right superficial temporal artery–middle cerebral artery bypass of patient A, demonstrating intraluminal embolic material traveling in a distal-to-proximal direction, observed on two occasions **(A,B)**.

**Figure 3:**
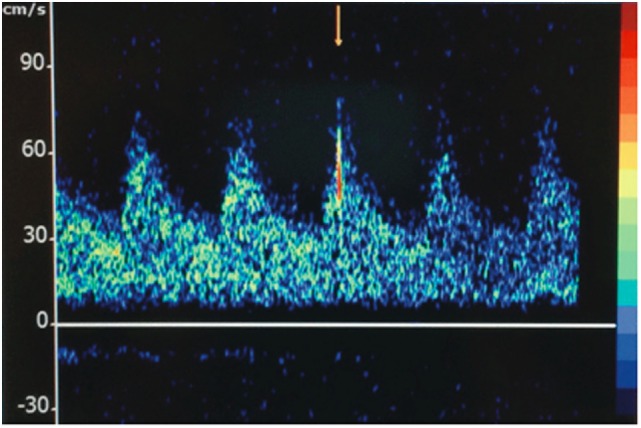
Transcranial Doppler displaying a high-intensity transient signal (HITS) in the right middle cerebral artery territory of patient A.

The STA parietal and frontal branches were used for anastomoses to two frontal MCA branches. On two occasions, embolic material was observed passing through the recipient artery in a distal-to-proximal direction (Figure 2; Video S1 in Supplementary Material). A portion was removed and sent for histological analysis, where it was interpreted as non-specific proteinaceous material (Figure S1 in Supplementary Material). The surgery was completed without radiographic or clinical evidence of new ischemia. On post-operative day 1, a surveillance TCD showed 8–10 HITS in the right MCA (Figure 3). While the patient was on aspirin, a platelet aggregation assay was suggestive of incomplete efficacy. Aspirin was increased from 81 mg daily to 325 mg daily, and 4 days later a repeat TCD study detected no HITS and the platelet aggregation study demonstrated improved platelet inhibition.

### Patient B

**Figure 4:**
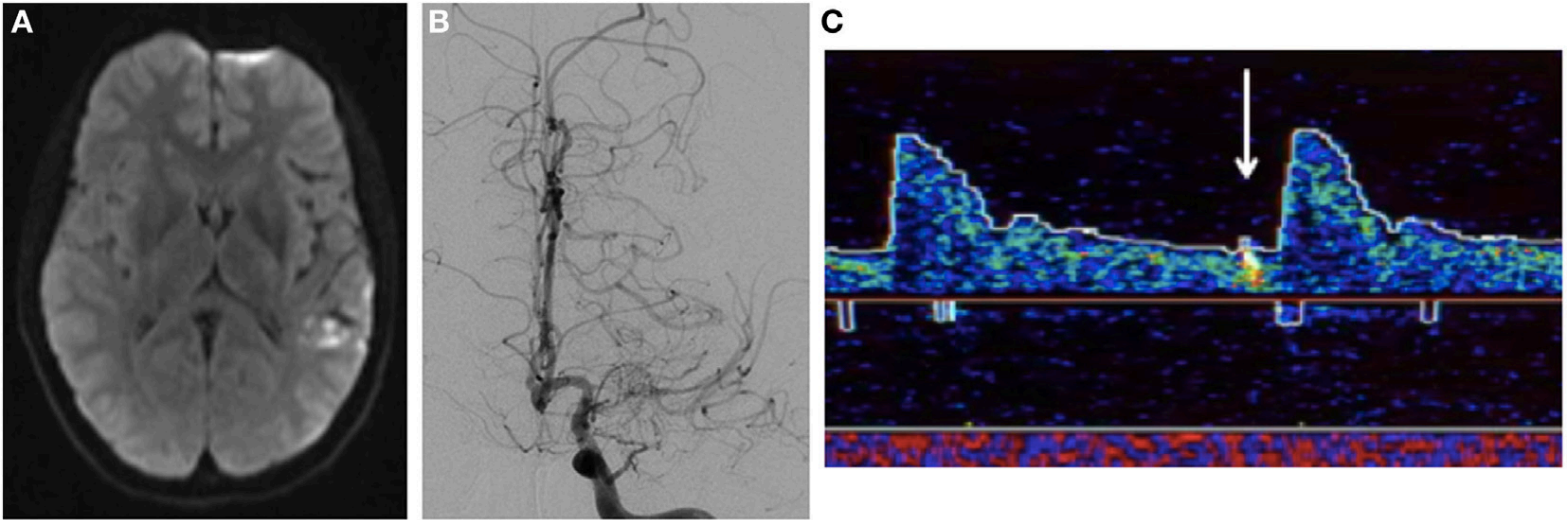
Patient B, a 37-year-old woman with left-sided moyamoya syndrome. **(A)** Magnetic resonance imaging demonstrates multiple embolic-appearing ischemic infarcts in the left middle cerebral artery (MCA) territory. **(B)** Cerebral angiogram with severe left ICA stenosis with collateralization. **(C)** Transcranial Doppler displaying HITS in the left MCA territory.

A 37-year-old woman with a history of complex migraines and Graves disease status post thyroidectomy presented with three weeks of progressive speech difficulty during the third trimester of pregnancy. On examination, she had a mild mixed aphasia. Brain MRI demonstrated embolic-appearing infarcts of various ages in her left frontal, temporal, and parietal lobes (Figure 4A). Conventional angiography showed occlusion of the left MCA M1 segment with local proliferation of collaterals and a normal right ICA (Figure 4B). Additional imaging was performed to assess the hemodynamic reserve of the left hemisphere and the patency of the left MCA. TCD vasoreactivity study demonstrated a lesser increase in flow velocity in the left compared with the right MCA at higher end-tidal carbon dioxide concentrations. A single-photon emission computed tomography (SPECT) study performed before and after 1,000 mg of intravenous acetazolamide showed baseline perfusion deficits in the left MCA territory with some areas of improved perfusion after the acetazolamide, suggestive of some degree of preserved autoregulation. Additional workup for etiology of stroke included a transesophageal echocardiogram, telemetry monitoring, and cerebrospinal fluid analysis, all of which were unrevealing. She had no history of radiation, atherosclerosis, or hyperlipidemia, and extensive serologic testing was unrevealing. Given her unilateral vasculopathy, negative secondary workup, and history of hyperthyroidism, she was diagnosed with moyamoya syndrome.

Transcranial Doppler demonstrated 26 HITS in the left MCA during a 30-min period (Figure 4C). When these HITS persisted in spite of therapeutic anticoagulation, aspirin 81 mg daily was added. After 3 months of treatment, no HITS were detected during a 40-min monitoring period. At that point, anticoagulation was discontinued, aspirin was continued, and she underwent a left STA–MCA bypass. She has remained well since the procedure on aspirin monotherapy, with a mild residual aphasia.

## Discussion

We present two patients with symptoms of cerebral ischemia and angiographic findings of moyamoya syndrome and disease with ultrasonic, visual, and pathological evidence of active brain embolization.

Hemodynamic compromise has long been considered the primary cause of ischemic symptoms in patients with moyamoya and is supported by several lines of evidence. Studies using xenon inhalation or single-photon positive emission computed tomography (SPECT) have shown impaired vasoreactivity distal to the stenosis, indicating that distal arterioles are maximally vasodilated and are unable to further augment flow (8, 11) Additionally, brain infarcts have a tendency to occur in areas of perfusion deficit seen on SPECT, in anatomically watershed distributions (12).

There is also clinical evidence to support the phenomenon of hypoperfusion. In children, there is an association between activities which reduce systemic carbon dioxide (such as crying) and subsequent cerebral vasoconstriction with transient ischemic symptoms (13). Furthermore, direct and indirect bypass operations which augment distal perfusion improve outcomes. The 5-year stroke rate of 12% (14) to 27% (9) in patients treated with any kind of bypass surgery is markedly improved compared with 40% (15) to 82% (16) in patients who did not have surgery. Although there is no randomized trial data to prove efficacy, surgery is considered the best available treatment for symptomatic moyamoya syndrome (17).

The possibility of embolism as an additional mechanism has also been recently investigated. In patients with proximal vascular stenoses, the two mechanisms can be difficult to separate and may coexist. One cannot rely solely on a typical watershed pattern of infarcts on imaging, as such infarcts have been associated with small distal vessels filled with cholesterol emboli (5). Both mechanisms may ultimately contribute to the infarcts, in that a state of chronic hypoperfusion from a proximal stenosis may prevent the distal washout of emboli. A TCD study may provide actionable information, as HITS have been associated with emboli in patients with proximal MCA stenoses, with number of HITS linearly correlating with number of infarcts on MRI (18).

In moyamoya disease, HITS are detected in about 12% (8) to 20% (9, 10) of patients. Their presence was predicted by recent clinical ischemic events and carried a corrected odds ratio of 10.6 for stroke in the next year (10). Furthermore, HITS are equally prevalent in all stages of the disease (10), a finding that might not be expected if hemodynamic compromise was the only factor at play.

The two patients discussed here provide convincing evidence of the presence of emboli in moyamoya patients. The nature and origin of this embolic material are more difficult to determine. While the pathological interpretation was non-specific, the fact that solid material was surgically extracted eliminates the possibility of the TCD and surgical findings being artifactual. It is also very unlikely that the embolus was a result of the procedure itself as it was seen traveling on two occasions in a distal-to-proximal direction, from an untouched arterial territory toward the surgical clamp. Possible sources of emboli include the severely stenosed proximal intracranial vessels, with disturbed hemodynamics explaining the reversal of flow in distal arteries. Alternatively, severe hemodynamic compromise from proximal stenosis could cause stasis in distal vessels with development of embolic material distally. Given that this finding was observed in both moyamoya disease (patient A) and moyamoya syndrome (patient B), we have no evidence to suggest that the risk of emboli formation would differ between these two groups.

This is the first report documenting direct visualization of brain embolism in moyamoya. In both cases, HITS were abolished with the use of antithrombotic therapy, but our data are insufficient to establish a cause and effect relationship.

## Conclusion

Hemodynamic compromise may not be the only cause of brain infarction in patients with moyamoya. Brain embolism occurs in this population, as seen here intra-operatively and by TCD, and may cause brain infarction. This provides a rational basis for antithrombotic therapy, which should be studied further.

## Ethics Statement

Please note that written informed consent was obtained from both patients for the publication of this report. As this is a case report, it was exempt from review by our local Institutional Review Board.

## Author Contributions

JS, primary physician for patient A, drafted manuscript, managed revisions, selected figures for patient A, and formatted all figures. SS, primary physician for patient B, made significant manuscript contributions, selected figures for patient B. HV, senior physician for patient B, supervised manuscript drafting and made revisions. VB: senior physician for patient A, supervised manuscript drafting and made revisions. NP, neurosurgeon for both patients, creator and owner of intra-operative photos and videos.

## Conflict of Interest Statement

HV is a paid consultant for Sage Therapeutics. VB receives funding from Boston Scientific (CEC member) and from Bayer (BMC-PI of a multi-center research study). None of these relationships influenced the work presented in this manuscript. The handling editor declared a shared affiliation, though no other collaboration, with the authors.

## Supplementary Material

Figure S1 Supplmentary Material figure, in TIFF format: Fragment of intraluminal cellular debris obtained during Patient A’s right STA-MCA bypass, noted to be nonspecific proteinaceous material.

Video S1 Supplementary Material video, in mp4 format: Intra-operative video captured during right STA–MCA bypass of patient A. On two occasions, direct visualization of emboli formation and distal-to-proximal passage is seen.
