# Supplementary material for: Direct Visualization of Arterial Emboli in Moyamoya Syndrome
Source: Front Neurol. 2017 Aug 24;8:425. doi: 10.3389/fneur.2017.00425 (PMC5609634; doi:10.3389/fneur.2017.00425)
Supplement: Figure S1 — Supplmentary Material figure, in TIFF format: Fragment of intraluminal cellular debris obtained during Patient A’s right STA-MCA bypass, noted to be nonspecific proteinaceous material. [file presentation_1.pdf]

Supplementary Material

Direct Visualization of Arterial Emboli in Moyamoya Syndrome

Julie G Shulman, MD[1*], Samuel Snider, MD[2], Henri Vaitkevicius, MD[2], Viken L Babikian, MD[1], Nirav J Patel, MD[3]

[1] Boston Medical Center, Department of Neurology, Boston University School of Medicine Boston, MA, USA
[2] Brigham and Women's Hospital, Department of Neurology, Harvard Medical School, Boston, MA, USA
[3] Brigham and Women's Hospital, Department of Neurosurgery, Harvard Medical School, Boston, MA, USA

***Correspondence:** Julie G. Shulman, MD: Julie.Shulman@BMC.org

Supplementary Material: *video, in mp4 format*:
Intra-operative video captured during right STA-MCA bypass of patient A. On two occasions, direct visualization of emboli formation and distal-to-proximal passage is seen.

This video can be accessed via Dropbox with the following link:
https://www.dropbox.com/s/tivwe5tnddf1zn2/Bypass%20with%20clots%20moyamoya%20with%20naration.mp4?dl=0

Supplementary Material: *figure, in TIFF format:*
Fragment of intraluminal cellular debris obtained during Patient A's right STA-MCA bypass, noted to be nonspecific proteinaceous material.

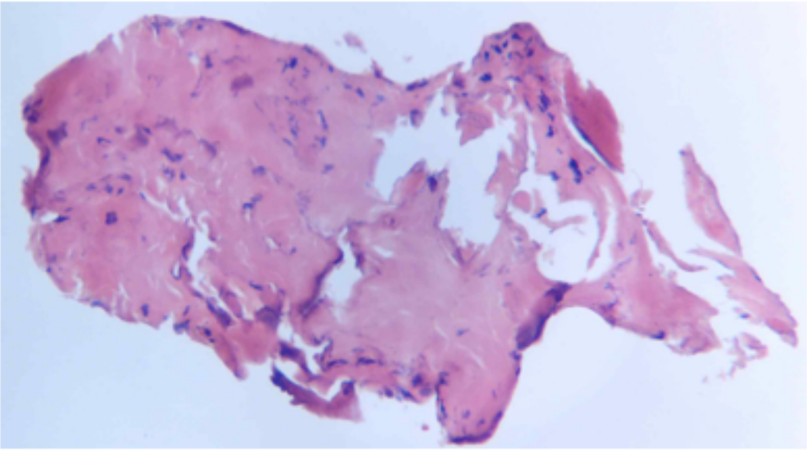

Supplemental Figure: Fragment of intraluminal cellular debris obtained during Patient A's right STA-MCA bypass, noted to be nonspecific proteinaceous material.
